# Brown Pine Leaf Extract and Its Active Component *Trans*-Communic Acid Inhibit UVB-Induced MMP-1 Expression by Targeting PI3K

**DOI:** 10.1371/journal.pone.0128365

**Published:** 2015-06-11

**Authors:** Won Bum Huh, Jong-Eun Kim, Young-Gyu Kang, Gaeun Park, Tae-gyu Lim, Jung Yeon Kwon, Da Som Song, Eun Hee Jeong, Charles C. Lee, Joe Eun Son, Sang Gwon Seo, Eunjung Lee, Jong Rhan Kim, Chang Yong Lee, Jun Seong Park, Ki Won Lee

**Affiliations:** 1 WCU Biomodulation Major, Center for Food and Bioconvergence, Department of Agricultural Biotechnology, Seoul National University, Seoul, Republic of Korea; 2 Advanced Institutes of Convergence Technology, Seoul National University, Suwon, Republic of Korea; 3 Skin Research Institute, Amorepacific Corporation R&D Center, Yongin, Republic of Korea; 4 Department of Food Science and Technology, Cornell University, Ithaca, New York, 14456, United States of America; 5 Traditional Alcoholic Beverage Research Team, Korea Food Research Institute, Seongnam, Republic of Korea; 6 Department of Biochemistry, King Abdulaziz University, Jeddah, Saudia Arabia; Institut national de la santé et de la recherche médicale - Institut Cochin, FRANCE

## Abstract

Japanese red pine (*Pinus densiflora*) is widely present in China, Japan, and Korea. Its green pine leaves have traditionally been used as a food as well as a coloring agent. After being shed, pine leaves change their color from green to brown within two years, and although the brown pine leaves are abundantly available, their value has not been closely assessed. In this study, we investigated the potential anti-photoaging properties of brown pine leaves for skin. Brown pine leaf extract (BPLE) inhibited UVB-induced matrix metalloproteinase-1 (MMP-1) expression to a greater extent than pine leaf extract (PLE) in human keratinocytes and a human skin equivalent model. HPLC analysis revealed that the quantity of *trans*-communic acid (TCA) and dehydroabietic acid (DAA) significantly increases when the pine leaf color changes from green to brown. BPLE and TCA elicited reductions in UVB-induced MMP-1 mRNA expression and activator protein-1 (AP-1) transactivation by reducing DNA binding activity of phospho-c-Jun, c-fos and Fra-1. BPLE and TCA also inhibited UVB-induced Akt phosphorylation, but not mitogen activated protein kinase (MAPK), known regulators of AP-1 transactivation. We additionally found that BPLE and TCA inhibited phosphoinositide 3-kinase (PI3K), the upstream kinase of Akt, in vitro. In summary, both BPLE and its active component TCA exhibit protective effects against UVB-induced skin aging. Taken together, these findings underline the potential for BPLE and TCA to be utilized as anti-wrinkling agents and cosmetic ingredients, as they suppress UVB-induced MMP-1 expression.

## Introduction

Human skin acts as an interface between the environment and our body [1]. Chronic ultraviolet (UV) exposure causes detrimental physiological changes to skin including epidermal inflammation, hyperpigmentation, immunosuppression and photoaging [2–5]. Among the subtypes of UV including UVA (320–400 nm), UVB (280–320 nm), and UVC (200–280 nm) [6], UVA and UVB are able to pass through the ozone layer and penetrate human skin. UVA and UVB make up 95% and 2–5%, respectively, of the UV radiation normally reaching skin [7]. In particular, UVB is the major source of skin damage and an etiological factor for many diseases and extrinsic skin aging as it has much higher intrinsic energy than UVA [8].

Photoaging, an extrinsic skin aging process, is characterized by pigmentary changes and severe wrinkle formation [9]. Exposure to UVB alters biological processes that promote matrix metalloproteinase (MMP) expression, decrease procollagen synthesis, and increase connective tissue damage [3, 9, 10]. Collagen breakdown by collagenase is a major cause of wrinkle formation. It is also related to extracellular matrix breakdown, which causes detrimental effects to connective tissues in various pathological situations [11–14]. MMP-1 is a critical enzyme for type-1 and type-3 collagen degradation in human skin [4, 8]. Preventing unbalanced collagen degradation via inhibition of MMP-1 expression represents a potential strategy for anti-photoaging.

UVB induces MMP-1 protein expression via the activation of cellular signaling. UVB irradiation activates cell surface receptors including the epidermal growth factor receptor (EGFR), which leads to propagation of intracellular signaling such as mitogen-activated protein kinases (MAPKs), phosphoinositide 3-kinase (PI3K)/Akt, Jak/STAT, and protein kinase-C [15–18] pathways. These pathways elevate activator protein 1 (AP-1) which is a transcription factor that plays a crucial role in transcription of MMP-1 [19]. The AP-1 family is composed of Jun proteins (c-Jun, v-Jun, JunB, JunD) and Fos proteins (c-Fos, v-Fos, FosB, Fra-1, Fra-2), involving activating transcription factors (ATF2, ATF3/LRF1, B-ATF) and basic region leucine zipper (bZIP) homodimerization or heterodimerization [20–22]. Phosphorylation of AP-1 subunits by their upstream kinases increases its translocation from the cytosol to the nucleus and enhances its DNA binding affinity. In turn, the target genes of AP-1 including MMP-1 are activated [23]. Therefore, by regulating upstream kinases such as PI3K/Akt or MAPK, target genes of AP-1 can be regulated.

Japanese red pine (*Pinus densiflora*), a member of the Pinaceae family, has long been cultivated in China, Russia, Japan, and Korea [24]. In Korea, approximately 65% of land is forest and pines occupy approximately 87% of the coniferous forests [25]. Pine leaves have historically been used as a food ingredient for color and flavor. They have also been used as a folk remedy for inflammatory diseases such as gastroenteritis, rheumatism, hemorrhage and asthma [26]. Fallen pine leaves stay green for 2 years until their color changes to brown [27]. Although these ‘brown pine leaves’ are abundant in forests and parks, they have not commonly been used or studied as a functional material.

In this study, we investigated the anti-photoaging effects of brown pine leaf extract (BPLE) and its active component *trans*-communic acid (TCA), for their potential application as anti-skin aging agents.

## Materials and Methods

### Chemicals and reagents

HaCaT cells were purchased from CLS Cell Lines Service GmbH (Eppelheim, Germany). Dulbecco’s modified eagle medium (DMEM) was purchased from Hyclone (Long, UT, USA). Fetal bovine serum (FBS) and β-actin antibody were bought from Sigma-Aldrich (St. Louis, MO, USA). The MMP-1 antibody was obtained from R&D Systems (Minneapolis, MN, USA). Antibodies against phosphorylated extracellular-signal regulated kinase 1/2 (ERK1/2) (Thr202/Tyr204), total ERK1/2, total c-Jun N-terminal kinase 1/2 (JNK1/2), phosphorylated-p38 (Thr180/Tyr182), and total p38 were purchased from Santa Cruz Biotechnology (Santa Cruz, CA, USA). Antibodies against phosphorylated Akt (Ser473), total Akt, phosphorylated JNK1/2 (Thr183/Tyr185), phosphorylated p90RSK (Thr359/Ser363) and total p90RSK were purchased from Cell signaling (Danvers, MA, USA). 3-[4,5-dimethylatiazol-2-yl]-2,5 diphenyltetrazolium bromide (MTT) powder was purchased from USB co. (Cleveland, OH, USA). Penicillin/streptomycin was purchased from Invitrogen (Grand Island, NY, USA). Protein assay kits were obtained from Bio-Rad Laboratories (Hercules, CA, USA).

### UVB irradiation

UVB (Bio-Link crosslinker, VilberLourmat, Cedex 1, France) was irradiated to the cells in serum-free media. The spectral peak of UVB was set at 312 nm. HaCaT cells were exposed to UVB at 0.01 J/cm^2^.

### Cell culture

HaCaT cells were cultured in 10% (v/v) FBS-DMEM with 1 μg/ml penicillin/streptomycin at 37°C under a 5% CO_2_ atmosphere. For the luciferase assay, HaCaT cells were stably transfected with AP-1 luciferase reporter plasmid. Transfected HaCaT cells were cultured in 10% (v/v) FBS-DMEM with 1 μg/ml puromycin.

### Human Skin equivalent preparation

Human skin equivalent (Neoderm^-^ED) was purchased from Tegoscience (Seoul, Republic of Korea). The human skin equivalent was treated with BPLE (5, 10 μg/ml) and TCA (5, 10 μM) for 1 h after 2 weeks of air-lift. The skin equivalent was then irradiated with UVB at 0.05 J/cm^2^ twice a day for 8 days, and the medium was changed every 2 days for 8 days. The skin equivalent was incubated at 37°C under a 5% CO_2_ atmosphere.

### Preparation of pine leaf extracts and isolation and purification of DDA and TCA

The green and brown leaves of *Pinus densiflora* Siebold & Zucc (pine needles) were collected from the Amorepacific Botanical Garden (Osan, Republic of Korea). Dried green pine needles and brown pine needles were ground and extracted with ten volumes of 80% ethanol for 2 days at room temperature. The extracts were obtained by filtering and evaporating the solvent. The brown pine needle extracts (11.4 g) were sequentially fractionated with hexane (HA), chloroform, butyl alcohol, and water. The HA fraction (2.93) was applied to a silica gel column (60, 70–230 mesh, Merck & Co., Whitehouse Station, NJ, USA) and eluted with chloroform and methanol solution (10:0.7 v/v) to obtain five fractions (Fractions Ι-V). Fraction ΙΙ was subjected to Rp-18 column chromatography (LiChroprep RP-18 25–40 μm, Merck & Co.) and eluted with 70% methanol. Compound II-A (348mg) and Compound II-C (826mg) were finally obtained as a single compound. A comparison of spectral data from compound ΙΙ-A analysis by several methods, including ^13^C nuclear magnetic resonance (NMR), ^1^H-NMR, and electron-ionization mass spectrometry (EI-MS) with data in the literature suggested the chemical structure to be DDA or abieta- 8,11,13- trien- 18- oic acid [28, 29]. Also, a comparison of spectral data from compound ΙΙ-C analysis by several methods, including ^13^C nuclear magnetic resonance (NMR), ^1^H-NMR, and electron-ionization mass spectrometry (EI-MS) with data in the literature (3) suggested the chemical structure to be TCA or 1,4a-Dimethyl-6-methylene-5-(3-methyl-penta-2,4-dienyl)-decahydro-naphthalene-1-carboxylic acid [30].

### Cell viability

Cell viability was measured by MTT assay. HaCaT cells were cultured in 96 well plates at a density of 4×10^5^ cells/well and were incubated in DMEM-10% FBS containing penicillin/streptomycin at 4°C in a 5% CO_2_ atmosphere. After reaching 80% cell confluence, the HaCaT cells were starved in serum-free DMEM for 24 h. The cells treated with different dosage of samples were incubated for 22 h at 37°C. After incubation, MTT solution was treated for 2 h. The medium was removed and the remaining formazan crystals in the cells were dissolved by DMSO. A microplate reader (Molecular Devices, Sunnyvale, CA, USA) was used to measure the color density at 570 nm.

### Real-time PCR

HaCaT cells (1.0×10^6^ cells in a 6 well dish) were treated with brown pine leaf extract and TCA for 15 h and harvested in RNAiso Plus (Takara Bio, Inc., Shiga, Japan). After reverse transcription with oligo-dT primers using a PrimeScriptTM 1st strand cDNA synthesis Kit (Takara Bio, Inc.), the cDNA was probed with the following primers (Bioneer, Daejeon, Korea): MMP-1 forward 5’-ATT CTA CTG ATA TCG GGG CTT TGA-3’, MMP-1 reverse 5’-ATG TCC TTG GGG TAT CCC TGT AG-3’ (409 bp); GAPDH forward 5’-GAG TCA ACG GAT TTG GTC GT-3’, GAPDH reverse 5’-TTG ATT TTG GAG GGA TCT CG-3’(517 bp). Before PCR amplification, the primers were denatured at 94°C for 5 min. Amplification consisted of 22 cycles: denaturation at 94°C for 30 s, annealing at 56°C for 1 min, and extension at 72°C for 1 min followed by a final 5 min extension at 72°C. PCR was performed with a Gene Amp PCR System 2700 (Applied Biosystems, Foster City, CA, USA). The RT-PCR reaction was performed using a CFX96 real-time PCR detection system (Bio-Rad). cDNA was amplified in the presence of iQ SYBR Green Supermix (Bio-Rad). To control for variations in mRNA concentration, all results were normalized to GAPDH. Relative quantitation was performed using the comparative ΔΔCt method following the manufacturer’s instructions.

### MMP-1 content measurement

Cultured HaCaT cells were starved in serum free-DMEM for 24 h to exclude potential FBS-activated cell signals. After starvation, the cells were treated with various concentrations of the samples for 1 h, followed by UVB (0.01 J/cm^2^) irradiation. The conditioned media was collected after 48 h of incubation, and the samples were analyzed with a DuoSet human total MMP-1 ELISA kit (R&D system Inc.) for MMP-1 content as described in the manufacturer’s protocols.

### Western blot assay

HaCaT cells were starved in serum free-DMEM for 24 h to lower the growth signals stimulated by FBS. After starvation, the cells were treated with various concentration levels of samples for 1 h, followed by UVB (0.01 J/cm^2^) irradiation. The cells were scraped in a lysis buffer [10 mM Tris (pH 7.5), 150 mM NaCl, 5 mM ethylene diamine tetra acetic acid (EDTA), 1% Triton X-100, 1 mM dithiothreitol (DTT), 0.1 mM phenylmethylsulfonyl fluoride (PMSF), 10% glycerol and protease inhibitor cocktail tablet], incubated on ice for 20 min, and then centrifuged at 18,620×g for 10 min. The protein concentrations were measured by a dye-binding protein assay kit (Bio-Rad) as described by the manufacturer. The proteins were separated by electrophoresis in a 10% SDS-polyacrylamide gel and transferred to Immobilon P membrane (Millipore, Billerica, MA, USA). The membrane was blocked with 5% fat-free milk for 1 h and then incubated with the specific primary antibody at 4°C overnight. Protein bands were visualized by a chemiluminescence detection kit (GE healthcare, NJ, USA) after hybridization with an HRP-conjugated secondary antibody (Santa Cruz Biotechnology).

### Ap-1 binding assay

Nuclear extracts preparation and the AP-1-family TransAM assay were performed following the manufacturer's instructions (Active Motif, Carlsbad, CA, USA).

### Zymography

Zymography was used to determine the activity of secreted MMP-2. Zymography was conducted with a 10% polyacrylamide gel in the presence of gelatin (0.5 mg/ml) as a substrate for MMP-2. The samples were suspended in loading buffer [10% SDS, 25% glycerol, 0.25 M Tris (pH 6.8) and 0.1% bromophenol blue], and then run on a 10% SDS-PAGE gel without denaturation. After electrophoresis, the gel was washed with renaturating buffer (Invitrogen) at room temperature for 30 min, and then incubated in developing buffer (Invitrogen) for 24 h at 37°C. The gel was then stained with 0.5% Coomassie brilliant blue.

### Lentiviral infection

The lentiviral expression vector pGF-AP1-mCMV-EF1-Puro was purchased from System Biosciences (Mountain View, CA, USA), and the packaging vectors, including pMD2.0G and psPAX, were purchased from Addgene Inc (Cambridge, MA, USA). A pGF-AP1-mCMV-EF1-Puro vector and the packaging vectors (pMD2.0G and psPAX) were transfected into HEK293T cells using jetPEI following the manufacturer's instructions. The transfection medium was changed at 4 h after transfection, and the cells were then cultured for 36 h. The viral particles were harvested by filtration using a 0.45 mm syringe filter, then combined with 8 μg/ml polybrene (EMD Millipore) and infected into 60% confluent HaCaT cells overnight. The cell culture medium was replaced with a fresh complete growth medium for 24 h before the cells were selected for using puromycin (2 μg/ml, Sigma-Aldrich) over 36 h. The selected cells were then used for further experiments.

### Luciferase reporter gene assay

HaCaT cells stably transfected with AP-1 luciferase plasmid were cultured in 96 well plates at a density of 4×10^5^ cells/well. After incubation in DMEM-10% FBS containing penicillin/streptomycin at 4°C in a 5% CO_2_ atmosphere, the cells were starved in serum-free DMEM for 24 h. Then the cells were treated with various concentration of samples for 1 h followed by UVB (0.01 J/cm^2^) irradiation. After 12 h of incubation, the cells were treated with lysis buffer [0.1 mM potassium phosphate buffer (pH 7.8), 2 mM ethylene diamine tetra acetic acid (EDTA), 1% Triton X-100, 1 mM dithiothreitol (DTT)] before their luciferase activities were measured by luminometer (Luminoskan Ascent; Thermo Electron, Helsinki, Finland).

### PI3K assay

To determine the PI3K activity in the presence or absence of BPLE and TCA, the HTRF assay was carried out by Eurofins Pharma Discovery (Dundee, UK). Briefly, various concentrations of BPLE and TCA were incubated with the recombinant p110α/p85α, p110β/p85α, and p110δ/p85α in the assay buffer. The reaction was initiated by addition of 200 μM ATP and stopped after 30 min of incubation by adding the stop solution containing EDTA and biotin-PIP3. Detection buffer was then added, and the resulting mixture was further incubated for 14 h. Signals from the wells were detected using the microplate reader. Time resolved fluorescence was measured at 620 nm and 665 nm after excitation at 337 nm and HTRF counts were determined according to the following formula HTRF = 10000*(Em 665 nm/Em 620 nm). The PI3K inhibitory activity of each compound was calculated according to the following formula: PI3K-inhibition (%) = (plusenzyme control–sample) / (plus-enzyme control–minus-enzyme control) × 100. For the plus-enzyme control, the kinase was incubated with PIP2 and ATP in the absence of BPLE or TCA, and for the minus-enzyme control, PIP2 was incubated with ATP in the absence of kinase and BPLE or TCA.

### Immunohistochemistry

Skin sections from the human skin equivalent were prepared for immunohistochemical staining of MMP-1 expression. Sections (5 mm thick) of 10% neutral formalin solution-fixed paraffin-embedded tissues were cut on silane-coated glass slides. They were deparaffinized three times with xylene, and dehydrated through a graded alcohol bath. The deparaffinized sections were incubated in citric acid buffer (pH 6.0) and boiled for antigen retrieval. To prevent nonspecific staining, each section was treated with 3% hydrogen peroxide for 10 min with a blocking solution containing 1% bovine serum albumin (BSA) for 30 min. For the detection of the MMP-1 target, the slides were incubated overnight with an affinity-purified primary antibody at 4°C in 1% BSA, and then developed using an anti-rabbit or anti-mouse Histostain Plus Kit (Zymed Laboratories Inc., South San Francisco, CA, USA). Peroxidase binding sites were detected by staining with 3,3’–diaminobenzidinetetrahydrochloride (Sigma-Aldrich). Mayer’s hematoxylin was applied as a counterstain (Sigma-Aldrich). Quantification was conducted using the Image J program with IHC tool box plug in.

### Masson’s trichrome staining

Skin sections from the human skin equivalents were prepared for Masson’s trichrome staining for collagen. Sections (5 mm thick) of 10% neutral formalin solution-fixed paraffin-embedded tissues were cut on silane-coated glass slides, and then deparaffinized three times with xylene and dehydrated through a graded alcohol bath. The deparaffinized sections were stained with hematoxylin for 5 min. The slides were then washed and stained in biebrich scarlet and acid fuchsin. Next, the slides were placed in phosphomolybdic-phosphotungstic acid for 10 min and aniline blue for 5 min to stain the collagen. The slides were then washed and incubated in 1% acetic acid for 15 min. Finally, they were dehydrated and washed. Quantification was performed using the Image J program with IHC tool box plug in.

### Statistical analysis

Data are expressed as the means ± standard deviation (S.D.). One-way analysis of variance (ANOVA) with Tukey’s HSD test was used to evaluate mean differences between groups and statistical significance. Differences were considered significant at *p*<0.05.

## Results

### BPLE has a stronger inhibitory effect against UVB-induced MMP-1 expression than PLE in HaCaT cells

**Fig 1:**
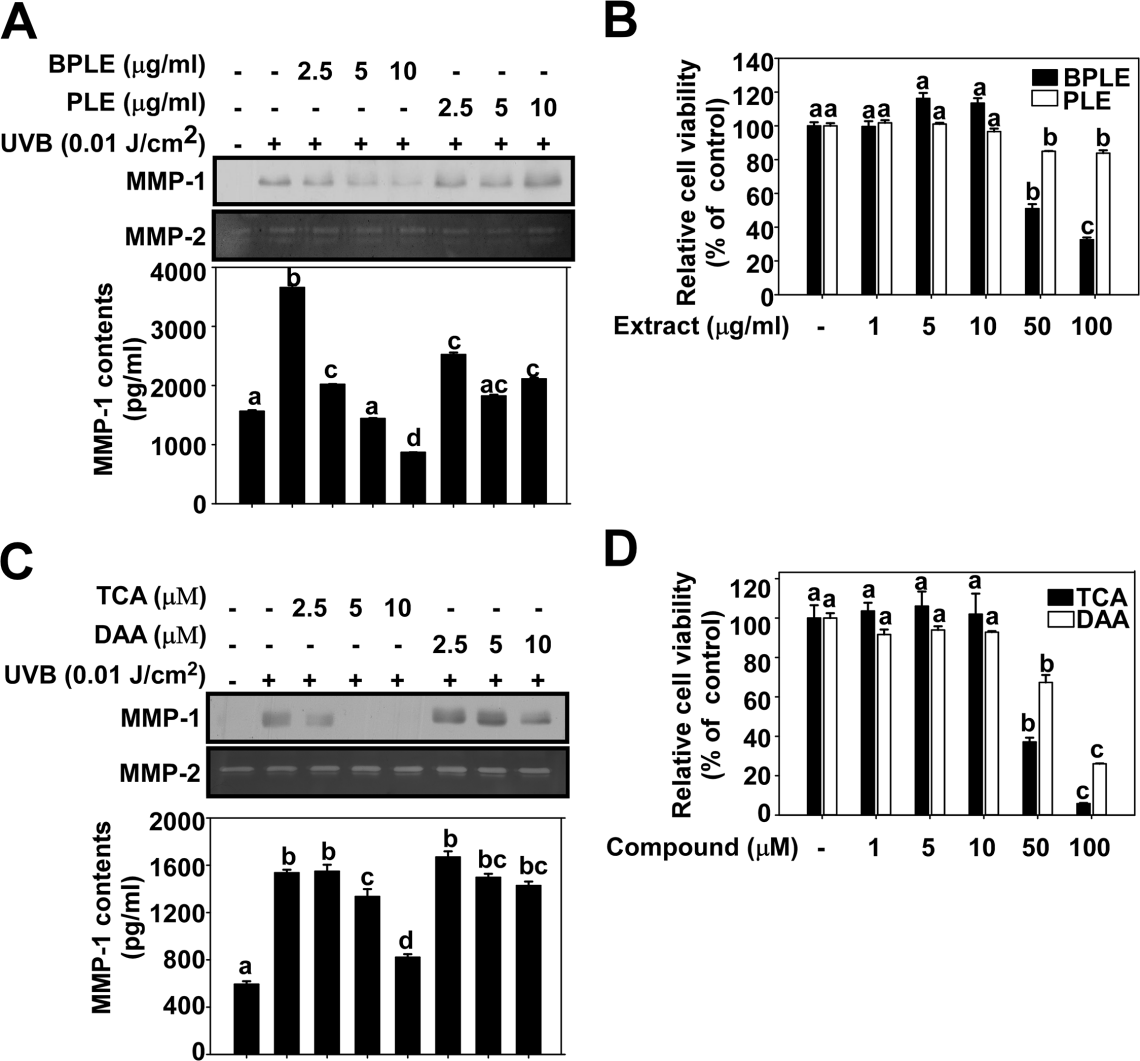
TCA contributes to the inhibitory effect of BPLE on UVB-induced MMP-1 expression in HaCaT cells. *A* and *C*, Protein expression was analyzed by Western blotting (MMP-1), Zymography (MMP-2), and ELISA (MMP-1 contents). Cells were treated with BPLE/PLE (A) or TCA/DAA (C) at the indicated concentration for 1 h before being exposed to 0.01 J/cm^2^ of UVB, and media was harvested 48 h later. *B* and *D*, Cell viability of HaCaT cells in the presence or absence of BPLE, PLE (B) or TCA, DAA (D). Cell viability was measured using MTT assay. Each experiment was performed in triplicate. The data are presented as the mean ±S.D. of MMP-1 protein content and cell viability. Means with different letters (a-c) within a graph were significantly different from each other at *p* < 0.05.

BPLE and PLE were tested for their inhibitory effect against UVB-induced MMP-1 expression in HaCaT cells within the range of concentration which did notexhibit cytotoxicity (Fig 1A and 1B). The results indicated that BPLE inhibits UVB-induced MMP-1 expression in a dose-dependent manner while not affecting MMP-2. This inhibitory effect of BPLE on MMP-2 was stronger than PLE, suggesting there is a compositional difference between BPLE and PLE which may result in their differential effects.

### TCA and DAA increase during the color change process of pine leaf from green to brown

Because BPLE has a markedly stronger inhibitory effect than PLE against UVB-induced MMP-1 protein expression, we hypothesized that the composition of pine leaves changes and new components with bioactive function are produced during the color changing process. To identify compositional difference between BPLE and PLE, we analyzed their chemical profile by HPLC. Notably, while *trans*-communic acid (TCA) and dehydroabietic acid (DAA) are detected as 90.5 mg/g and 14.26 mg/g each in PLE, their quantities went up to 180.62 mg/g and 60.3 mg/g each in BPLE (S1 and S2 Figs). This finding leads us to further test the effect of TCA and DAA in HaCaT cells.

### TCA is a key bioactive component in BPLE

To verify if TCA and DDA contribute to the bioactive function of BPLE, the effect of TCA and DAA against UVB-induced MMP-1 protein expression in HaCaT cells were examined at concentrations that were non-cytotoxic (Fig 1C and 1D). The results showed that TCA, but not DAA, inhibited UVB-induced MMP-1 protein expression in a dose-dependent manner. Therefore, TCA is thought to be a major active component of BPLE, which may be, at least partially, responsible for the activity of BPLE.

### BPLE and TCA inhibits UVB-induced MMP-1 mRNA expression and AP-1 transactivation

**Fig 2:**
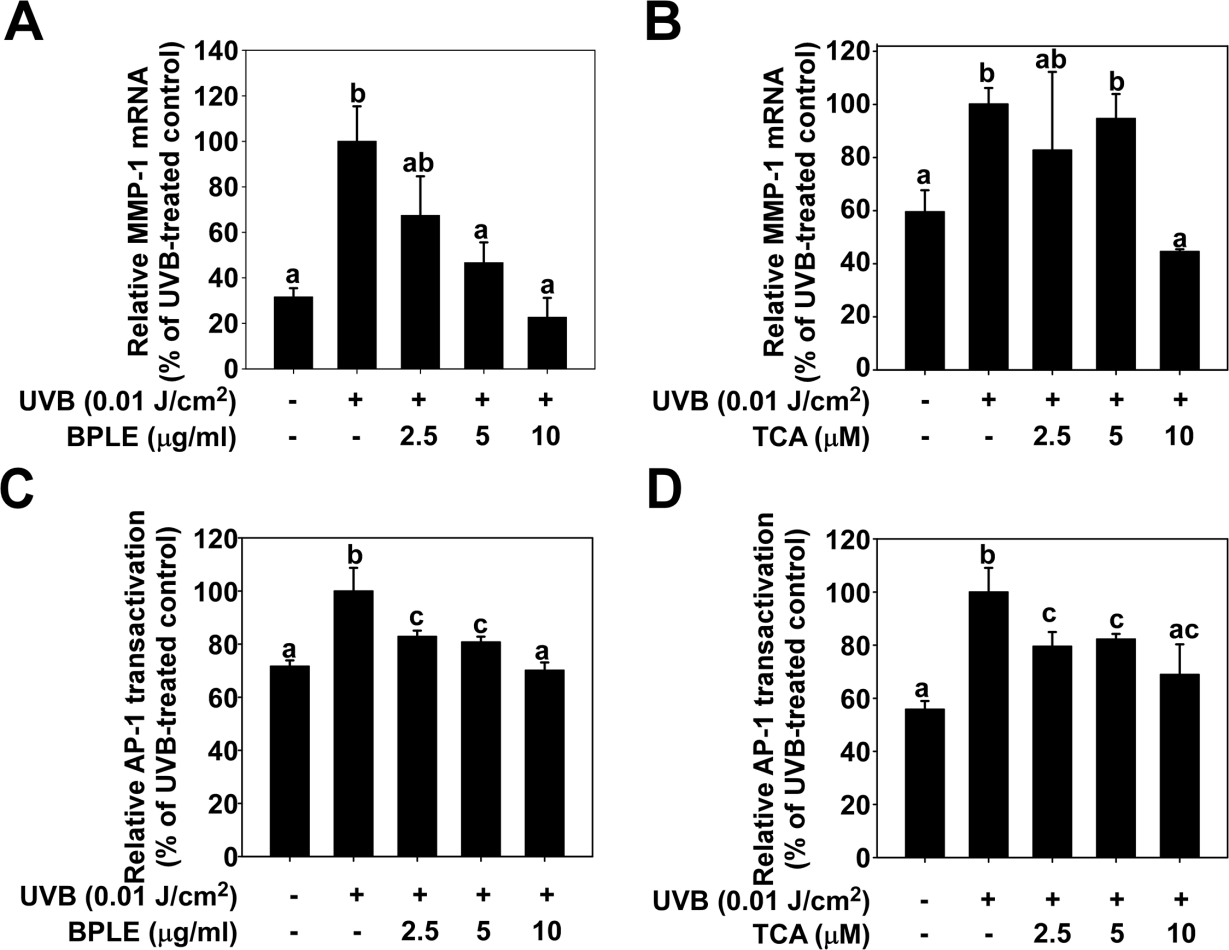
BPLE and TCA inhibit UVB-induced MMP-1 mRNA expression and AP-1 transactivation in HaCaT cells. *A and B*, Cells were treated with BPLE and TCA at the indicated concentrations for 1 h before being exposed to 0.01 J/cm^2^ of UVB and mRNA was collected 12 h later. MMP-1 mRNA expression was analyzed as qRT-PCR. *C and D*, BPLE and TCA attenuated UVB-induced AP-1 transactivation in HaCaT cells. Cells were treated with BPLE and TCA at the indicated concentration for 1 h before being exposed to 0.01 J/cm^2^ of UVB and cells were lysed 12 h later. AP-1 transactivation was measured using Luciferase assay. All data are presented as the mean ±S.D. determined from three independent experiments. Means with different letters (a-c) within a graph were significantly different from each other at *p* < 0.05.

We further examined the effect of BPLE and TCA on MMP-1 mRNA expression, and both BPLE and TCA reduced UVB-induced MMP-1 mRNA levels (Fig 2A and 2B). It has been reported that AP-1 is a major transcription factor of UVB-induced MMP-1 expression [3, 31–33]. To verify whether AP-1 mediates the effect of BPLE and TCA, AP-1 transactivation was examined using HaCaT cells stably transfected with AP-1 luciferase plasmid. The results showed that both BPLE and TCA attenuated UVB-induced AP-1 transactivation (Fig 2C and 2D) suggesting that AP-1mediates MMP-1 regulation by BPLE and TCA.

### BPLE and TCA suppresses UVB-induced binding affinity of AP-1 residues

**Fig 3:**
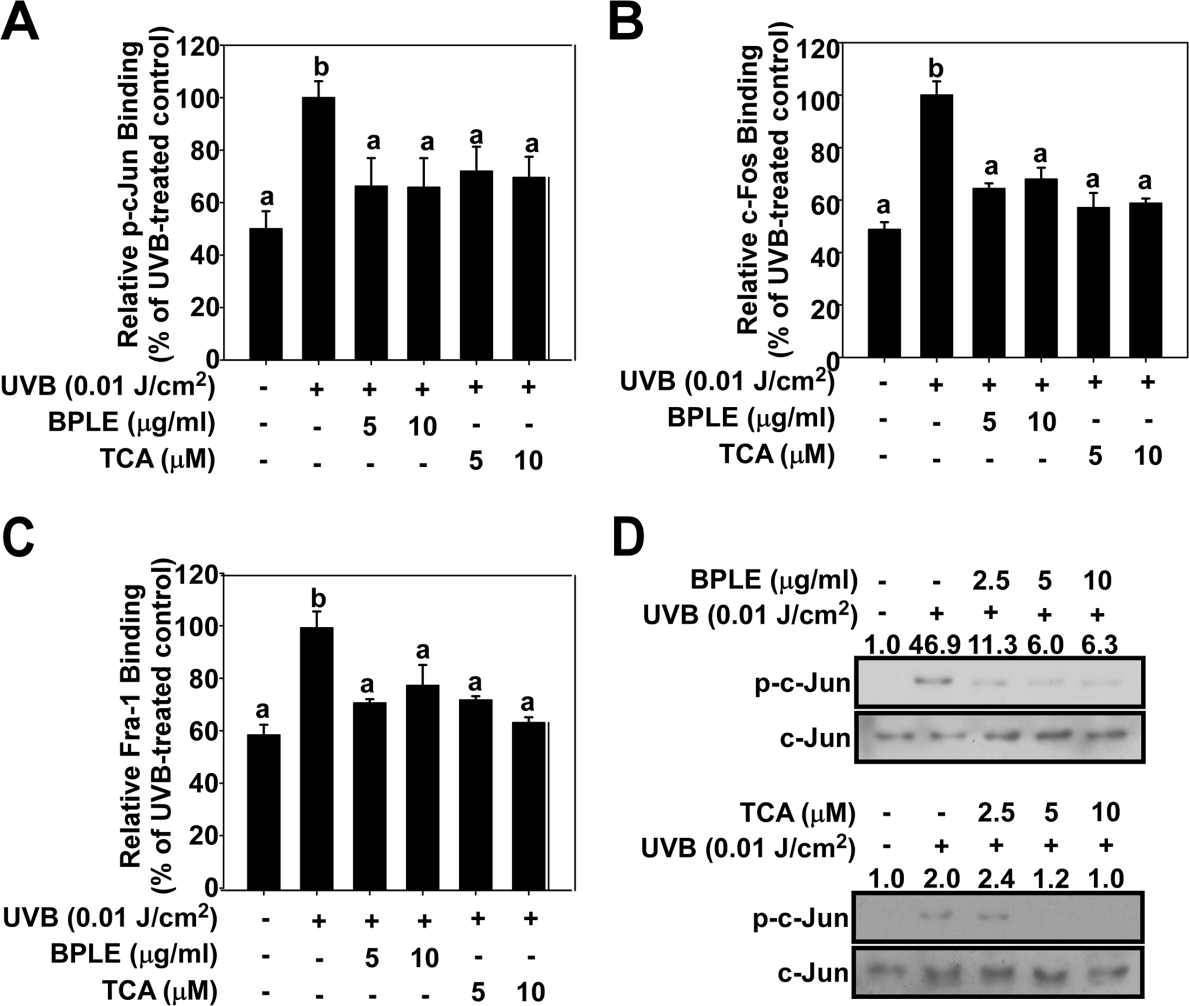
BPLE and TCA inhibit UVB-induced binding affinity of AP-1 residues in HaCaT cells. *A*, *B*, *and C*, DNA binding activity of phospho-c-jun (A), c-Fos (B) and Fra-1 (C). Cells were treated with BPLE and TCA at the indicated concentrations for 1 h before being exposed to 0.01 J/cm^2^ of UVB and nuclear extract was collected 4 h later. DNA binding activity was measured by TransAM AP-1 DNA-binding ELISA kit. Means with different letters (a-d) within a graph were significantly different from each other at *p* < 0.05. *D*, Phosphorylated and total protein levels of c-Jun were measured by Western blot assay. Relative band intensity is expressed as fold of control and indicated on top of each band.

To identify which AP-1 residues are affected by BPLE and TCA, we measured binding affinity of AP-1 subunits by TransAM AP-1 DNA-binding ELISA kit. The binding affinity of each subunit phospho-c-Jun (Ser73), c-Fos, and Fra-1 was increased by UVB. Both BPLE and TCA inhibited UVB-induced p-c-Jun (Ser73), c-Fos, and Fra-1 DNA binding affinity (Fig 3A, 3B and 3C). The Western blot analysis confirmed that BPLE and TCA inhibited UVB-induced p-c-Jun (Ser73, Fig 3D). Therefore, BPLE and TCA inhibit MMP-1 expression by reducing the DNA binding affinity of AP-1 residues including c-Jun, c-Fos, and Fra-1.

### BPLE and TCA inhibits PI3K activity in vitro

**Fig 4:**
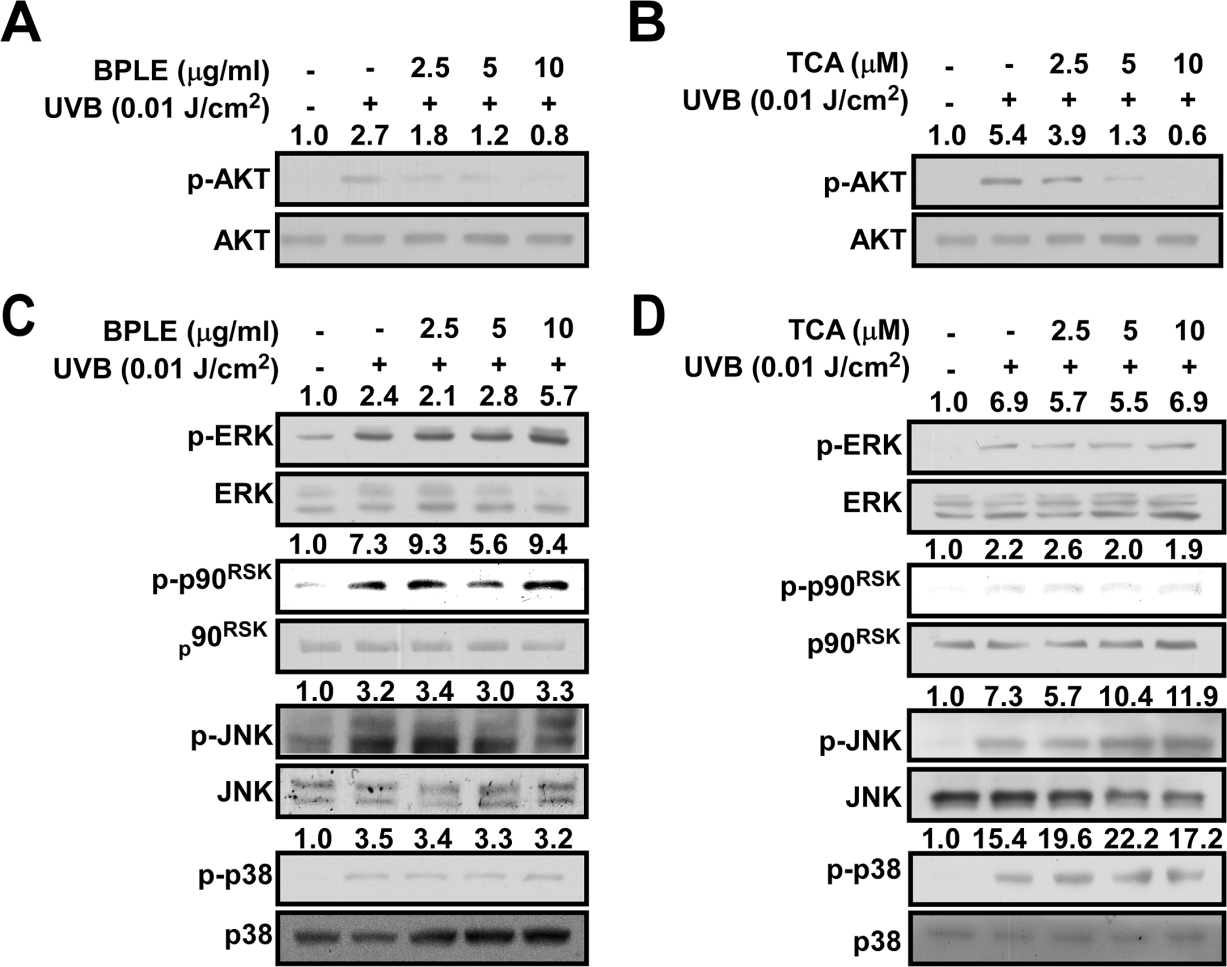
BPLE and TCA inhibit UVB-induced Akt phosphorylation but not Mitogen-activated kinase (MAPK) signaling pathway in HaCaT cells. *A-D*, Cells were treated with BPLE (A, C) and TCA (B, D) at the indicated concentration for 1 h before exposed to 0.01 J/cm^2^ of UVB and collected after 30 min. Phosphorylated and total protein levels were analyzed by Western blot assay. Relative band intensity is expressed as fold of control and indicated on top of each band.

**Fig 5:**
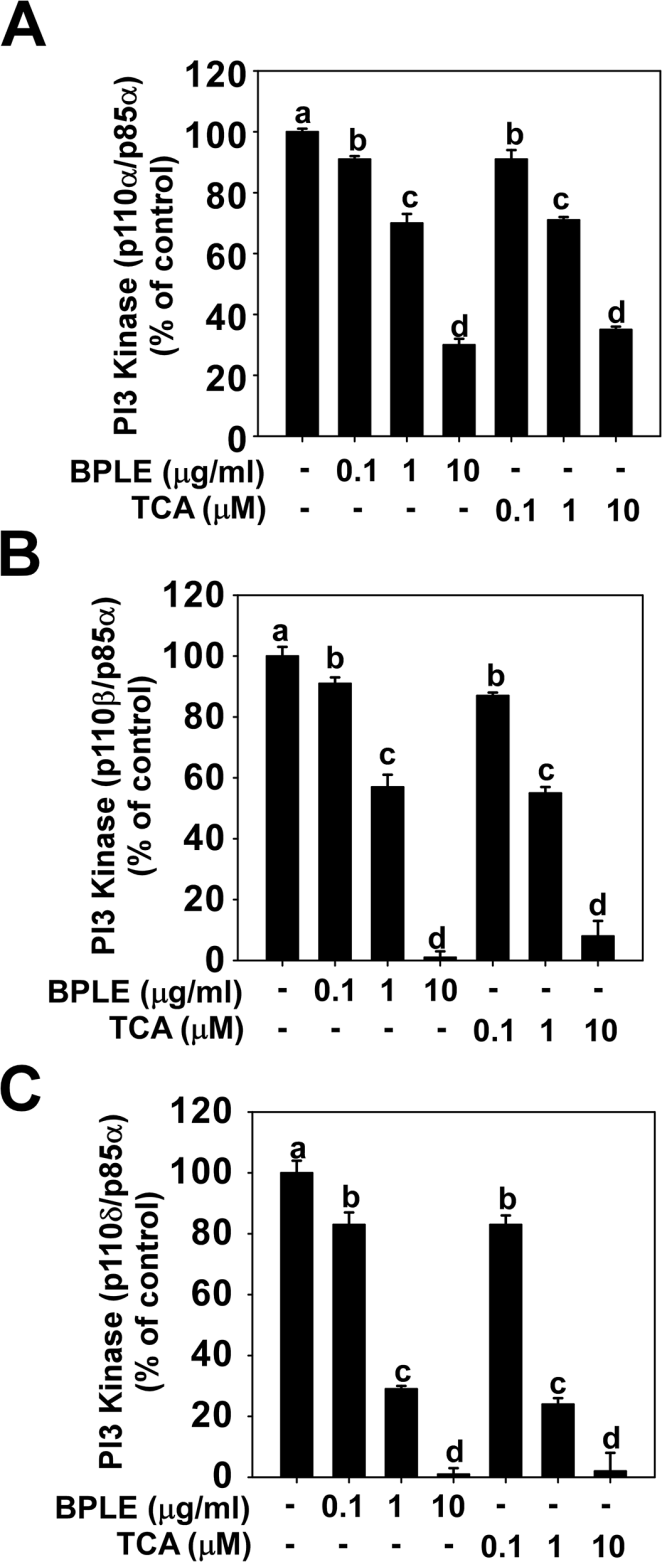
BPLE and TCA inhibit Phosphoinositide 3-kinase (PI3K) activity. *A*, *B and C*, PI3K activity was measured by HTRF described in the Materials and Methods. PI3K isoforms, p110α/p85α (A), p110β/p85α (B), and p110δ/p85α (C) were used for measuring activity. Means with different letters (a-d) within a graph were significantly different from each other at *p* < 0.05.

PI3K/Akt and MAPK cellular signal pathways play a crucial role in UVB-induced AP-1 transactivation [16, 34, 35]. We monitored the effect of BPLE and TCA on the phosphorylation of PI3K/Akt and MAPK pathways. Our results revealed that BPLE and TCA suppressed Akt phosphorylation (Fig 4A and 4B), but not MAPK including ERK, p90RSK, JNK, and p38 (Fig 4C and 4D). Subsequently, we investigated whether PI3K, an upstream kinase of Akt, could be targeted by BPLE and TCA. Indeed, we found that BPLE and TCA inhibited the kinase activity of three isoform types of PI3K (p110α/p85α, p110β/p85α, and p110δ/p85α) in a dose-dependent manner *in vitro* (Fig 5).

### BPLE and TCA reduce collagen degradation and attenuate MMP-1 expression in a human skin equivalent model

**Fig 6:**
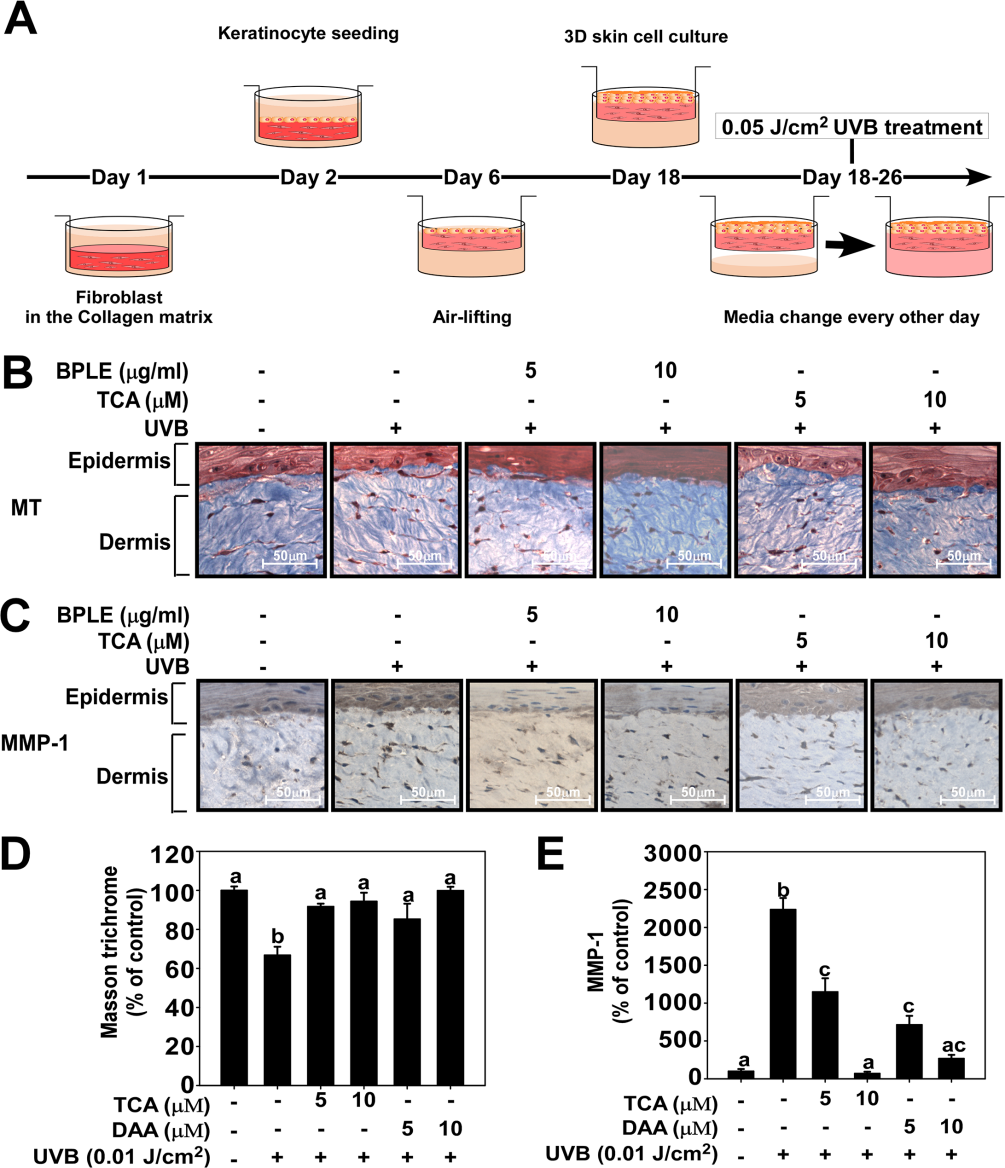
BPLE and TCA decrease collagen degradation and MMP-1 expression in a human skin equivalent model *A*, A schematic diagram of the 3D human skin cell culture system. The experimental procedure was described in the Materials and Methods. *B and C*, BPLE and TCA inhibit UVB-induced MMP-1 protein expression levels and collagen degradation. The serial sections, from the human skin equivalent, were mounted onto silane-coated slides and subjected to Masson’s trichrome staining (B and D) or to immunohistochemical staining using anti-MMP-1 antibody (C and E). MMP-1 appears brown. Relative density was measured using the ImageJ program. Means with different letters (a-d) within a graph were significantly different from each other at *p* < 0.05.

To further investigate the anti-photoaging effect of BPLE and TCA in physiological conditions, a human skin equivalent model was employed. The skin equivalent system was generated as described in the Materials and Methods (Fig 6A). To examine the effect of BPLE and TCA in the skin equivalent system, UVB-induced collagen degradation and MMP-1 overexpression were monitored after Masson’s trichrome staining and immunohistochemical staining, respectively. Our results showed that BPLE (5, 10 μg/ml) and TCA (5, 10 μM) have a protective effect against UVB-induced collagen degradation (Fig 6B and 6D) and MMP-1 overexpression (Fig 6C and 6E) in this model.

## Discussion

An increasing number of studies have reported the health benefits of natural botanical compounds, and recently their effects on skin health, especially skin aging, have been gaining a great deal of interest. Recent studies show protective effects of various dietary polyphenols and botanical extracts including citrus and rosemary extract [36], almond phytochemicals [37], and coffee extract [38] against UV-induced photoaging. However, the molecular mechanism underlying the photoprotective effects of the natural compounds has not been investigated in-depth. To aid the utilization of naturally abundant and effective botanical sources as cosmetic material, in-depth mechanistic studies and the identification of molecular targets as well as clinical data should be considered.

TCA and DAA are major compounds that increase in production as pine leaves changed their color. Only TCA (and not DAA) exhibits inhibitory effects against UVB-induced MMP-1 expression. TCA represents a group of diterpenic acids with a labdane skeleton containing three double bonds and a carboxyl group. There are five communic acids that differ in the location of the double bonds and the orientation of the carboxyl group. Notably, TCA is the most abundant communic acid found in nature. It is widely distributed in Cupresaceae species, especially in the genus Juniperus [39]. Previous studies have reported that it exhibits antimycobacterial [40], antitumoral [41, 42], anti-inflammatory, and antioxidant [43] activities. In this study, we showed that TCA is a dominant compound in BPLE and exerts inhibitory effects on UVB-induced MMP-1 expression, similar to BPLE. In addition, we showed that both BPLE and TCA exert their effect through a similar molecular mechanism. Therefore we suggest that TCA is the major active component that enhances the functionality of BPLE compared to PLE.

Both BPLE and TCA inhibit UVB-induced MMP-1 mRNA and AP-1 transactivation. AP-1 is a major transcription factor in UVB-induced MMP-1 expression [3, 31–33] and it binds to the TPA-response element (TRE) and activates the transcription of target genes including MMP-1 [20, 44]. AP-1 is composed of homodimers or heterodimers of Jun or heterodimers of Jun-Fos proteins [23]. Both BPLE and TCA inhibited UVB-induced DNA binding activity of c-Jun, c-fos and Fra-1. UVB-induced c-Jun phosphorylation is also inhibited by BPLE and TCA treatment. MAPK and PI3K/Akt pathways are the major regulators of DNA binding activity of AP-1 [45–48]. BPLE and TCA inhibited UVB-induced Akt phosphorylation, but not MAPK phosphorylation. Therefore, it can be concluded that BPLE and TCA inhibit UVB-induced MMP-1 expression via the PI3K/Akt pathway.

The PI3K/Akt pathway plays a crucial role in regulating various cellular processes including inflammation and aging processes by modulating a number of genes [49]. PI3K generates phosphatidylinositol (3,4,5)-trisphosphate (PIP3) at the plasma membrane. Akt binds PIP3 through its pleckstrin homology (PH) domain, resulting in translocation of Akt to the membrane and is activated by phosphorylation though PDK1 and mTOR-rictor complex [50]. The PI3K/Akt pathway is induced by UVB and mediates various cellular responses including MMP-1 expression [49]. Therefore, blocking PI3K represents a potential strategy for preventing the harmful effects of UVB. In this study, we found that BPLE and TCA inhibit three isoforms of PI3K (p110α/p85α, p110β/p85α, and p110δ/p85α). Therefore, inhibition of UVB-induced Akt phosphorylation by BPLE and TCA stems from the inhibition of PI3K activity by BPLE and TCA. This also underlines the potential use of BPLE and TCA as pan-PI3K inhibitors.

The human skin equivalent model is a type of three dimensional cell culture system, which is suggested to overcome the limits of two dimensional cell culture systems. Three dimensional cell culture systems are often used to study diseases such as netherton syndrome [51], ichthyosis [52] and skin inflammation [53]. The model carries clinical relevance, and is free from concerns for animal welfare. Our results show that UVB-induced collagen degradation and MMP-1 expression decrease in the BPLE- and TCA-treated human skin equivalent. These results suggest the potential effectiveness of BPLE and TCA if used in a clinical setting.

In summary, our results demonstrated that BPLE has a stronger inhibitory effect on UVB-induced MMP-1 expression than PLE in human keratinocytes, and that TCA, a major active component of BPLE, reproduces this effect of BPLE. BPLE and TCA attenuated UVB-induced AP-1 transactivation and Akt phosphorylation through direct inhibition of PI3K activity. Furthermore, the protective effect of BPLE and TCA against UVB-induced collagen degradation and MMP-1 expression were confirmed in the human skin equivalent model. Clinical evidence may further unveil its potential to be developed as a valuable cosmeceutical material.

## Supporting Information

S1 Fig Isolation and characterization of TCA from BPLE and PLE. BPLE and PLE (0.02 g) was prepared in ethanol (80%). Isolation was conducted as described in Materials and Methods. BPLE appears as green and PLE is blue.
(TIF)

S2 Fig Isolation and characterization of DAA from BPLE and PLE. BPLE and PLE (0.02 g) was prepared in ethanol (80%). Isolation was conducted as described in Materials and Methods. BPLE appears as green and PLE is blue.
(TIF)

S3 Fig A schematic diagram for the protective mechanism of BPLE and TCA against UVB-induced skin photoaging. (TIF)
