# Supplementary figures and images for: Brown Pine Leaf Extract and Its Active Component Trans-Communic Acid Inhibit UVB-Induced MMP-1 Expression by Targeting PI3K
Source: PLoS One. 2015 Jun 11;10(6):e0128365. doi: 10.1371/journal.pone.0128365 (PMC4465834; doi:10.1371/journal.pone.0128365)

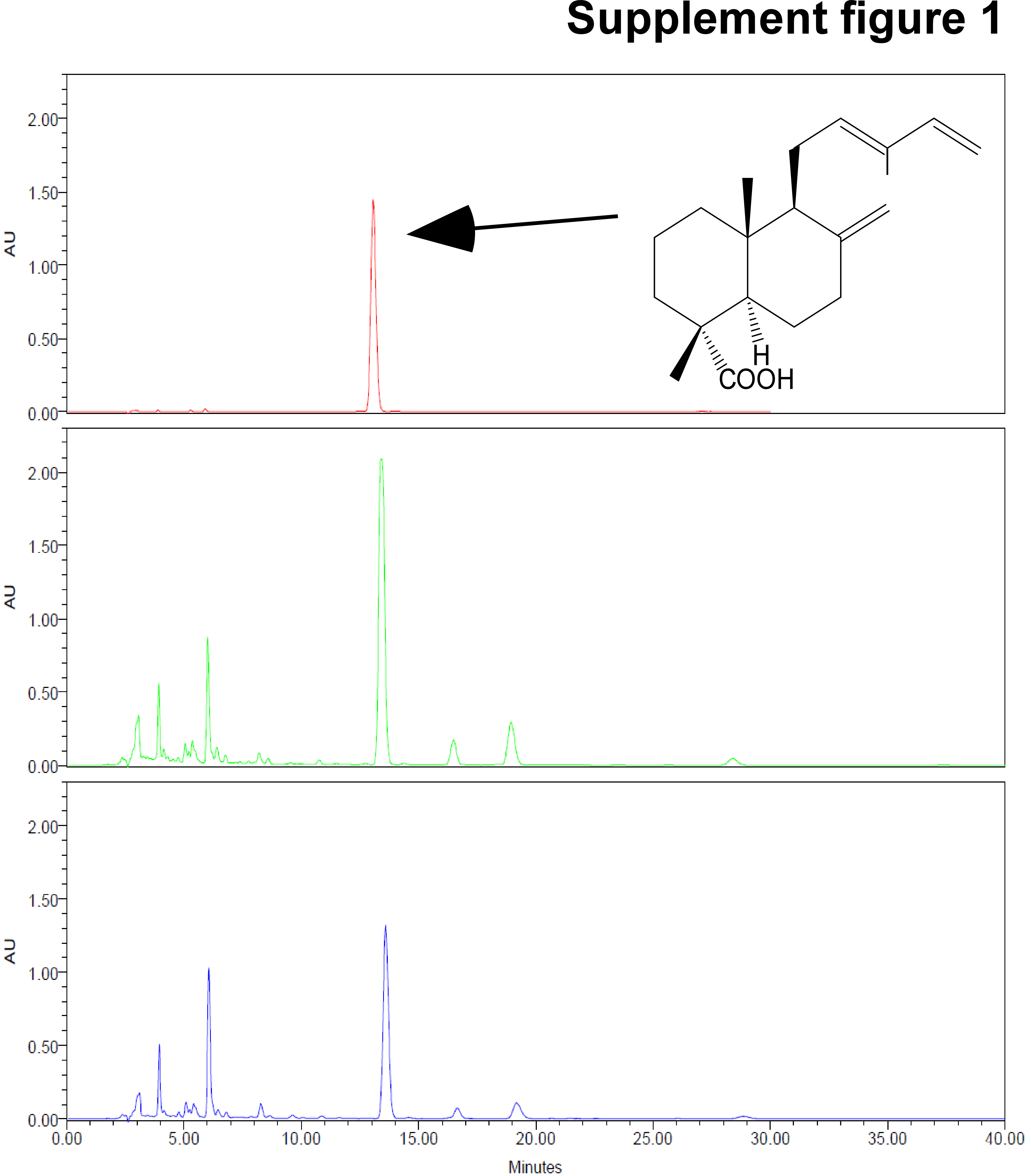

Supplement: S1 Fig — BPLE and PLE (0.02 g) was prepared in ethanol (80%). Isolation was conducted as described in Materials and Methods. BPLE appears as green and PLE is blue. (TIF) [file pone.0128365.s001.tif]

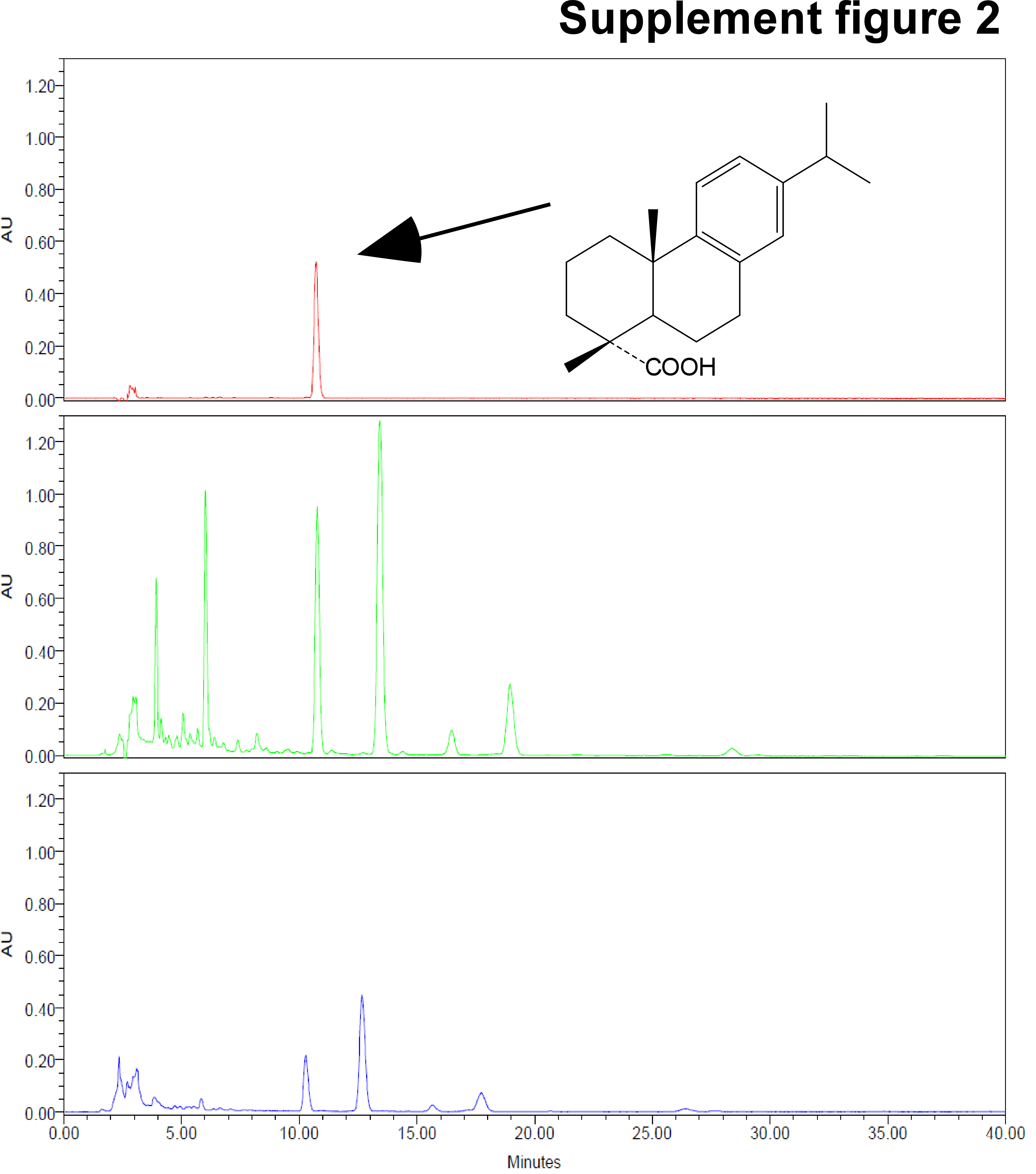

Supplement: S2 Fig — BPLE and PLE (0.02 g) was prepared in ethanol (80%). Isolation was conducted as described in Materials and Methods. BPLE appears as green and PLE is blue. (TIF) [file pone.0128365.s002.tif]

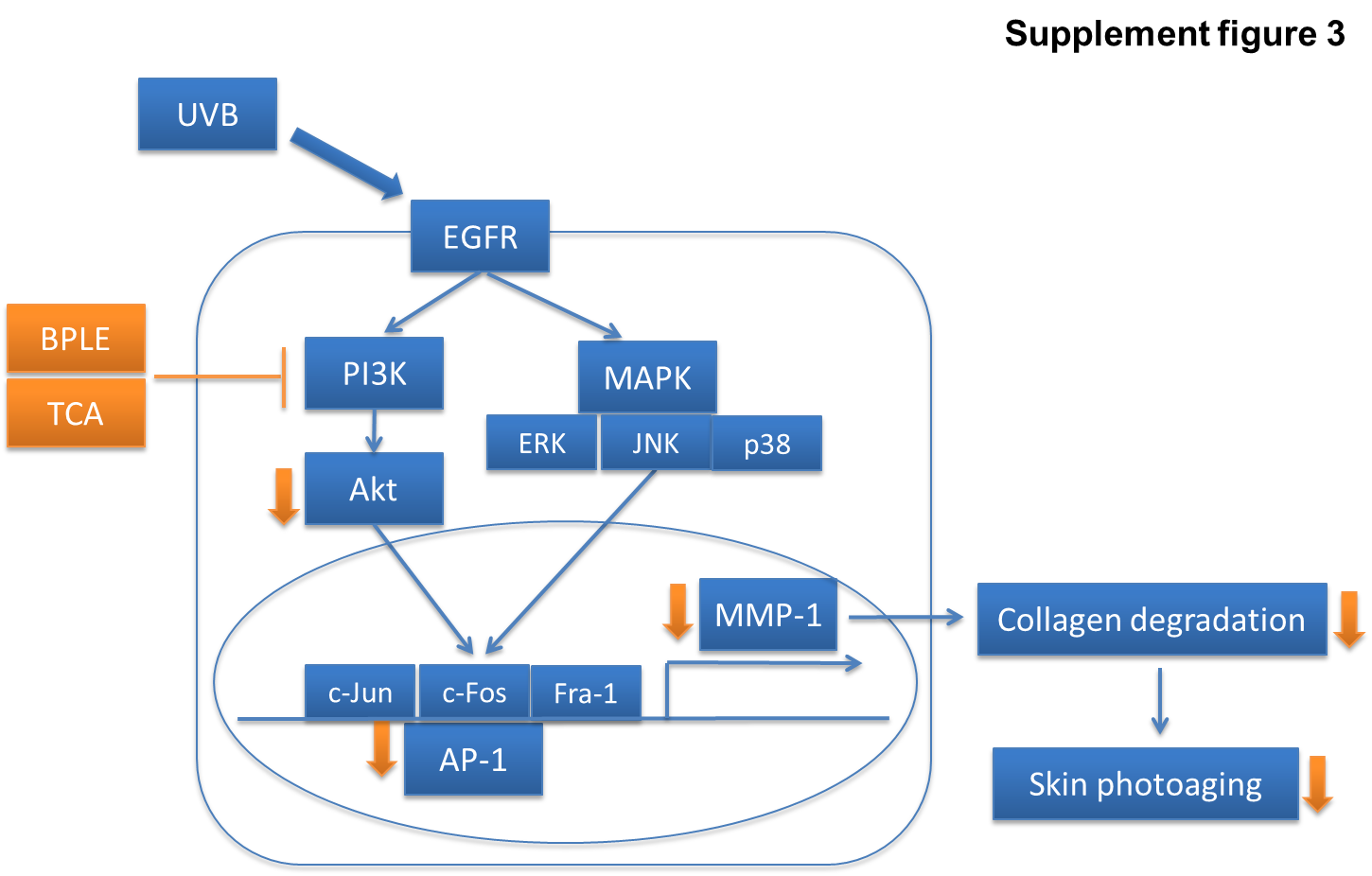

Supplement: S3 Fig — (TIF) [file pone.0128365.s003.tif]
